# SMARCA4 (BRG1)-deficient carcinoma invading the skull base: report of two cases and literature review

**DOI:** 10.1097/MS9.0000000000002158

**Published:** 2024-05-15

**Authors:** Hongji Zhu, Ying Liu

**Affiliations:** Department of Radiology, The First Affiliated Hospital of USTC, Division of Life Sciences and Medicine, University of Science and Technology of China, Hefei, Anhui, China

**Keywords:** carcinoma, computed tomography, magnetic resonance imaging, skull base, SMARCA4 (BRG1)-deficient

## Abstract

**Introduction and importance:**

SMARCA4 (BRG1)-deficient carcinomas in the head and neck are a rare and highly aggressive group of malignant tumors. They lack typical clinical and imaging features and are often misdiagnosed.

**Case presentation:**

The authors report two male patients with a history of smoking. Case 1 presented with nose bleeding as the first symptom, whereas case 2 presented with headache with blurred vision. Preoperative computed tomography (CT) and MRI suggested a highly aggressive malignant tumor of the head and neck with invasion of the skull base. Case 1 could not be operated on because of the large size of the punctured tumor. Case 2 underwent the surgery. The final pathological diagnosis was SMARCA4 (BRG1)-deficient carcinoma. At the 6-month follow-up, case 1 died. After completing the full course of chemotherapy, case 2 reported progressively worsening headaches and hearing loss.

**Discussion and conclusion:**

SMARCA4 (BRG1)-deficient carcinoma in the head and neck is a rare and highly aggressive malignant tumor that is advanced at diagnosis, prone to invasion of adjacent structures, difficult to operate on, and has a poor prognosis. CT and MRI play a vital role in evaluating the size and extent of the tumor, invasion of adjacent structures, and distant metastasis. It provides a significant reference for clinical diagnosis and therapeutic decision-making. Different patients of SMARCA4 (BRG1)-deficient carcinoma in the head and neck respond differently to radiotherapy and chemotherapy. Early use of next-generation sequencing (NGS) or immunohistochemistry(IHC) techniques is helpful in guiding treatment planning.

## Introduction

Highlights

- SMARCA4 (BRG1)-deficient carcinoma is a rare clinical disease; both cases invade the structures of the skull base and might be utilized for physicians' reference and knowledge.
- The two cases had similar pathological types, but the imaging presentations had similarities and differences. Correct recognition and diagnosis by radiologists can help clinicians choose the proper treatment.
- Different patients of SMARCA4 (BRG1)-deficient carcinoma in the head and neck respond differently to radiotherapy and chemotherapy.

SMARCA4 (BRG1)-deficient carcinomas are a rare group of highly aggressive tumors that can occur in multiple sites, such as the nasal cavity, paranasal sinuses, thorax, lungs, gastrointestinal tract, and ovaries. SWI/SNF-deficient sinus carcinomas were included in the latest WHO classification of head and neck neoplasms as a separate category in 2022^1^. The SWI/SNF family of complexes is an essential eukaryotic chromatin remodeling complex, and mutations in its subunits ( SMARCB1/INI1 and  SMARCA4/BRG1) are associated with various human diseases. In the head and neck region, SMARCB1-deficient carcinomas are rare and mainly involve paranasal sinuses, especially sieve ones. However, SMARCA4-deficient carcinomas are much rarer and mainly involve the nasal cavity^2^. It accounts for 4% of malignant tumors of poorly differentiated epithelial origin in the sinuses and is prevalent in males with a median age of less than 50 years^3^. In this study, we retrospectively analyzed two cases of SMARCA4-deficient carcinoma in the head and neck with skull base invasion, reviewed and discussed clinical and radiologic features, histologic findings, and the treatment options of the disease in order to enrich the spectrum of cancers with nasal sinus SMARCA4 deletion and improve the understanding and identification of this disease.

## Case presentation

**Figure 1:**
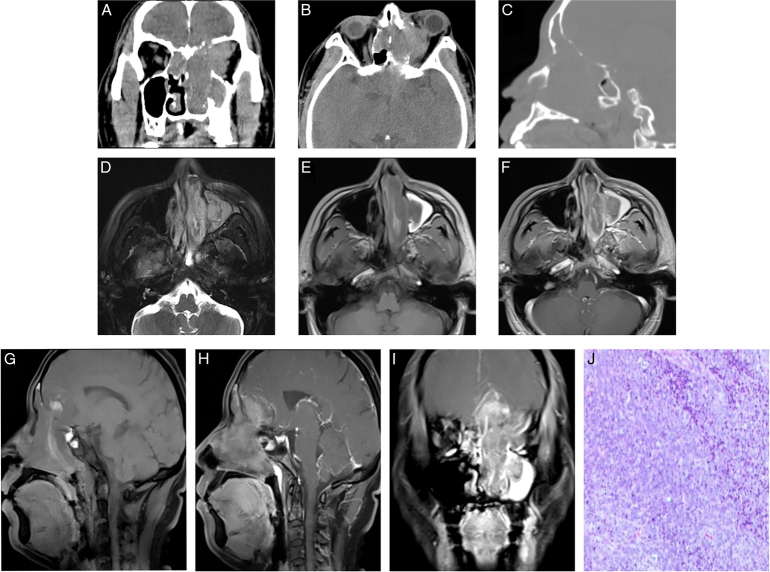
CT and MRI examinations. (A–C ) CT images showed a massive occupation in the left nasal cavity, invading the left maxillary sinus, breaching the orbital plate to the left orbit, invading the intracranium upwards through the sieve bone horizontal plate, and worm-eaten bone destruction at the base of the skull. (D–I) MR images showed short T1 signals of strips in the lesion, suggesting that the tumor was combined with hemorrhage. The lesion was heterogeneous and intensified after enhanced, invading the nasopharynx backward and the intracranium upward through the anterior skull base, (J) Microscopically, the tumor cells were densely distributed in diffuse patches, with rounded and oval cells, obvious heteromorphisms, easy-to-see nuclear schizophrenic images, and interstitial stroma with patchy necrosis. CT, computed tomography.

Case 1: A 34-year-old male was admitted to our hospital with intermittent bleeding from the left nasal cavity for 1 month. Physical examination revealed that the patient had a left-sided nasal tumor blocking the nostril, and the eye movement on the left side was limited. A huge mass was observed in the left nasal cavity on computed tomography (CT) and MR images, invading the left maxillary sinus and left orbit and moving upward into the anterior skull base to the intracranium. (Fig. 1A-I). No distant metastases were found on preoperative examination. Initially, our diagnosis was olfactory neuroblastoma because of the location of the tumor in the olfactory region at the base of the anterior cranial fossa. The diagnosis of SMARCA4 (BRG1))-deficient carcinoma was confirmed by biopsy. IHC showed INI1 (+), BRG1 (−), Ki-67 (+70%)(Fig. 1J). After preoperative multidisciplinary consultation, the patient was treated with chemotherapy (docetaxel 120 mg d^1^ + cisplatin 40 mg d1^-3^) because of the large tumor size and invasion of the base of the skull, which was difficult to resect. After the first course of chemotherapy, the patient underwent TOMO radiotherapy. The patient developed liver damage, bone marrow suppression, and pulmonary infection. Supportive therapy was administered, including hepatoprotective, platelet-boosting, anti-infective, and electrolyte-correcting treatments. Unfortunately, there was no improvement in the patient's condition. The patient also showed signs of pupil dilatation and retardation of light reflex and then was discharged with abandonment of treatment. At 1-month follow-up after discharge, the patient had died.

**Figure 2:**
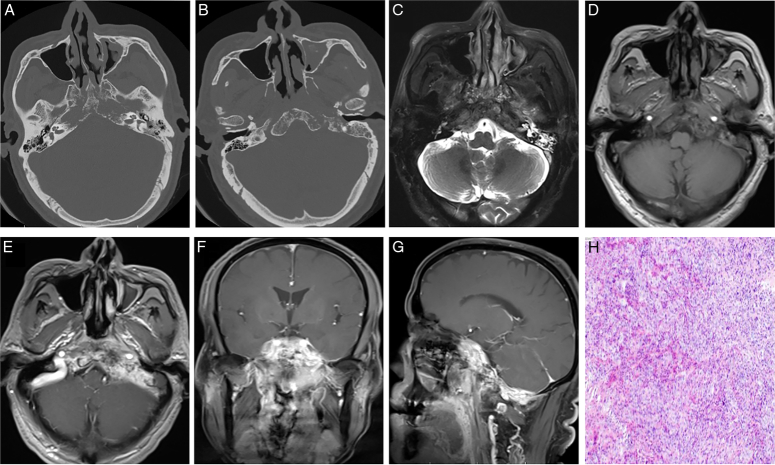
CT and MRI examinations. (A, B) Diffuse destruction of bone in the sphenoid bone, bilateral petrous apex, left temporal bone mastoid, occipital bone, and slope on CT imagings. (C–G) It showed marked thickening of the surrounding meninges enhanced on MR imagings. (H) Microscopically, tumor cells infiltrated within fibrous tissue with prominent atypia and easily visible mitotic images. CT, computed tomography.

Case 2: A 64-year-old man was admitted to the hospital with left-sided headache, hearing loss, and blurred vision for more than half a year. Physical examination showed smooth mucosa in the oropharynx, new biological protrusions on the right side of the uvula, and no obvious abnormalities in the nasopharynx; diffuse destruction of bone in the sphenoid bone, bilateral petrous apex, left temporal bone mastoid, occipital bone, and slope, with marked thickening of the surrounding meninges enhanced on CT and MR imaging (Fig. 2A-G). No distant metastases were found on preoperative examination. At first, we diagnosed the tumor as a chordoma because it was mainly located on the slope at the base of the middle cranial fossa. During surgery, multiple bone invasions were observed in the skull base. Additionally, a significant amount of neoplasm was detected on the facial nerve and cranial fossa surfaces with the appearance of fish flesh, and it was challenging to resect it completely. Pathological analysis indicated a SMARCA4 (BRG1))-deficient carcinoma (Fig. 2H). IHC revealed INI1 (+), BRG1 (−), and Ki-67 (+; ~40%). The patient received eight chemotherapy courses every three weeks after surgery, respectively, docetaxel 120 mg/day and carboplatin 600 mg/day. During the follow-up periods of 1, 3, and 6 months, he presented with progressively worsening headaches and hearing loss.

## Discussion

SMARCA4 (BRG1)-deficient carcinomas comprise a group of neoplastic lesions that share the common feature of SMARCA4 gene de-expression, which is relatively rare and can occur at different sites, such as the central nervous system, head and neck, thorax, gastrointestinal tract, and female reproductive system^4^. They are reported to have some standard clinicopathological features, such as undifferentiated small round cells or rhabdomyosiform morphology, highly invasive features, high malignancy, and poor prognosis^5–7^.

SMARCA4-deficient carcinomas in the head and neck are poorly reported in the literature, most of which are case reports. Parsel *et al.* ^8^ reviewed all patients with SMARCB1-deficient cancers of the nasal sinuses reported in the literature and pointed out that the prognosis of this type of tumor was poor and that most of the tumors were in the advanced stage, with a tendency to metastasize at the time of discovery. Zhongyu and colleagues reported 16 cases of SMARCB1-deficient carcinoma of the nasal sinus in China. They concluded that the site of development of this type of tumor is the nasal sieve sinus^9^. It was easy to invade the adjacent orbital wall and anterior cranial base with an invasive bony reaction, consistent with the image manifestation of case 1 in this paper.SMARCB1 (INI1) and SMARCA4 were in the SWI/SNF complex. The pathogenic mechanism of these tumors is that the gene inactivation mutations or deletions lead to the deletion of the corresponding gene products^10–13^. Schaefer and colleagues suggested that all genes of the SWI/SNF complex, except SMARCB1 and SWI/SNF subunits other than SMARCA4, are rarely involved in head and neck malignancies, and SMARCA4-deficient sinus carcinomas have demographic and clinical features similar to those of SMARCB1-deficient sinus carcinomas. Males are preferred; the median age is lower (~44 years), and the nasal cavity is the leading site of involvement, followed by multiple combined sinus sites^14^. Nonetheless, it has also been documented that SMARCA4 deletion carcinomas are more aggressive than SMARCB1 deletion sinus carcinomas, with 2/3 of patients dying from the disease within one year^15,16^. The first that needs to be identified is undifferentiated carcinoma of the nasal sinuses, originating from the mucosal epithelium, prone to involve multiple adjacent sinuses and the skull base, with extensive bone destruction, unclear borders, and frequent intratumoral necrosis, which is difficult to identify on imaging. Amigay *et al.* ^3^. studied 10 cases of SNUC specimens, all with complete deletion of the tumor suppressor gene SMARC4, and they detected that they had in common the IDH2 mutation. Undifferentiated carcinomas of the nasal sinuses and SMARC4 complete deletion tumors are believed to be a relatively homogeneous group^17^. Another type of malignant tumor that needs to be identified is neuroendocrine carcinoma. It can occur anywhere in the nasal sinuses and is highly invasive. Necrosis is common, and invasion along the lymphatic vessels is also joint. The tumor can also invade the skull base, and its characteristic feature is a marked enhancement on MRI. Immunohistochemistry can help identify the most sensitive and specific markers, such as CgA, Syn, and INSM1^18^. The similarities between cases 2 and 1 were that both SMARCA4-deficient carcinomas invaded the skull base, and both were male patients with a history of smoking. However, the central part of the tumor in case 1 was in the nasal cavity and invaded the base of the anterior cranial fossa. In contrast, the central part of the tumor in Case 2 was located at the base of the middle cranial fossa. Little is known about the invasion of the middle cranial fossa in SMARCA4 (BRG1)-deficient carcinomas. Reinhard *et al.* ^19^ reported a case of SMARCA4 (BRG1)-deficient carcinoma invading the middle cranial fossa of the right pontocerebellar horn region. SMARCA4(BRG1) is a deficient carcinoma that invades the brainstem and middle skull base. In addition, Duan *et al.* ^20^ reported cases of poorly differentiated chordomas of the SMARCB1/INI1 deletion type, with all five cases occurring in the slope region of the middle skull base with varying degrees of intracranial invasion. This was similar to the presentation of case 2 in this study, a patient who was preoperatively diagnosed with chordoma. Case 2 also needs to be differentiated from chondrosarcoma, which can also occur at the base of the skull and is aggressive. However, his characteristic presentation is the large amount of calcification that can be seen on the CT image, which was not found in this case.

The definitive diagnosis relies primarily on pathologic examination. The most important feature of SMARCA4 (BRG1)-deficient carcinomas was the complete absence of SMARCA4 in tumor cells in all cases, but INI1 expression was preserved.

Epithelial markers may be partially expressed or not. Tumor cells are usually positive for vimentin, but in a few cases, it is not expressed. Neuroendocrine markers Syn may be weakly positive in focal areas, thus predisposing to misdiagnosing large cell neuroendocrine carcinoma, but CgA is usually not expressed. CK7 and CK20 may be reduced or absent.

A large tumor with intracranial invasion was found in case 1. Unfortunately, it was not possible to resect the tumor through surgery. The patient underwent a course of radiation and chemotherapy, but a severe adverse reaction to the treatment resulted in the patient's death within a month of discharge from the hospital. In case 2, the patient underwent tumor reduction surgery and received the complete course of chemotherapy. Taxane in combination with platinum was the regimen in the two cases series, similar to primary treatment options for head and neck malignancies in other studies^21^. However, the response and efficacy of the treatment varied significantly between the two patients. We hypothesized that this chemotherapy regimen might not be suitable for all SMARCA4-deficient carcinoma in head and neck; this has been confirmed in previous research. Kang *et al.* ^22^ reported some patients underwent NGS testing that revealed CD274 amplification, PD-L1 overexpression, and response to ICIs (immune checkpoint inhibitors, ICIs). However, a larger sample size is necessary to fully validate and elucidate the treatment method for this rare disease.

## Conclusion

The study reported two cases with standard features, such as being male and smokers. The imaging results showed that the skull base was invaded by malignant tumors, which caused worm-like bone destruction. The location of this destruction differed between the anterior and middle cranial fossa. In the future, when encountering head and neck tumors that invade the skull base, radiologists should focus on evaluating the tumor size, scope, and adjacent structures. They should also consider SMARCA4 (BRG1)/SMARCB1-deficient carcinoma as a possible diagnosis to expand their diagnostic options. This will help improve the accuracy of imaging and provide a strong basis for clinical decision-making and surgical planning. Different patients of SMARCA4 (BRG1)-deficient carcinoma in the head and neck respond differently to radiotherapy and chemotherapy. Early use of next-generation sequencing (NGS) or immunohistochemistry (IHC) techniques is helpful in guiding treatment planning.

## Ethical approval

Not applicable.

## Consent

Written informed consent was obtained from the patient for publication of this case report and accompanying images. A copy of the written consent is available for review by the Editor-in-Chief of this journal on request.

## Source of funding

Not applicable.

## Author contribution

H.Z. wrote the original manuscript; Y.L. guided writing and reviewed the manuscript.

## Conflicts of interest disclosure

There are no conflicts of interest.

## Research registration unique identifying number (UIN)

Not applicable.

## Guarantor

Hongji Zhu.

## Data availability statement

The data that support the findings of this study are available from the corresponding author upon reasonable request. Some data may not be made available because of privacy or ethical restrictions.

## Provenance and peer review

Not applicable.
